# Effects of slaughter weight on carcass characteristics, meat quality, and metabolomics profiling in the *longissimus dorsi* muscle of Tianfu finishing pigs

**DOI:** 10.3389/fvets.2024.1420634

**Published:** 2024-06-28

**Authors:** Yuanfeng Li, Xuan Tao, Pinyao Zhao, Jianchuan Zhou, Xiang Ao

**Affiliations:** ^1^School of Life Sciences, Liaocheng University, Liaocheng, Shandong, China; ^2^Faculty of Quality Management and Inspection & Quarantine, Yibin University, Yibin, China; ^3^Animal Breeding and Genetics Key Laboratory of Sichuan Province, Sichuan Animal Science Academy, Chengdu, China; ^4^Solid-state Fermentation Resource Utilization Key Laboratory of Sichuan Province, Yibin, China; ^5^Sichuan Higher Education Engineering Research Center for Agri-Food Standardization and Inspection, Yibin, China; ^6^School of Animal Science and Technology, China Agricultural University, Beijing, China; ^7^Sichuan Techlex Industrial Co. Ltd., Mianyang, China

**Keywords:** slaughter weight, metabolomics, meat quality, Tianfu finishing pig, *longissimus dorsi* muscle

## Abstract

In order to investigate the effect of slaughter weight (SW) on carcass characteristics and meat quality, we measured the carcass characteristics, meat quality, and amino acid metabolomics characteristics of *longissimus dorsi* (*LD*) muscle from Tianfu finishing (TF) pigs. Based on SW, 13 pigs were divided into three groups (100-kg group, 125-kg group, and 150-kg group with 3, 5, 5 pigs in each group, respectively). Raising SW to 125 kg or 150 kg increased average backfat thickness (*P* < 0.01) and intramuscular fat content (*P* < 0.01), and decreased shear force (*P* < 0.01). A total of 231 amino acid metabolome from three amino acid classes identified with metabolomics were analyzed, and 93 differentially expressed metabolites (DEMs) were identified (69 up-regulated DEMs and 24 down-regulated DEMs). The DEMs, including urea, 3-iodo-L-tyrosine, N-glycyl-L-leucine, and N, N-dimethylglycine with amino acid metabolism, were significantly induced (*P* < 0.01). KEGG pathway analysis showed that these DEMs were significantly enriched (*P* < 0.01) in 135 metabolism pathways, including pathways related to amino acid metabolism, such as arginine and proline metabolism, glycine, serine and threonine metabolism, alanine, aspartate and glutamate metabolism, tryptophan metabolism, and beta-alanine metabolism. Our research findings provided new insights into the impact of SW on amino acid distribution and theoretical support for genetic breeding of meat quality of TF pigs. However, raising SW to 125 kg, or more, decreased the carcass leanness of live TF pigs and had no benefits to pork quality attributes.

## 1 Introduction

The attention paid to the quality of animal protein products is constantly growing (1). As pork is the most consumed animal protein, enhancing its quality is of great significance for the development of animal husbandry (2, 3). The factors that determine meat quality include pig breed, feeding mode, growth rate, and feed nutrients (4–10). Among these factors, the slaughter weight (SW) of finishing pigs has a significant impact on meat quality. Optimizing the timing of sales not only reduces production costs but also meets consumer demands for high-quality pork.

Tianfu finishing (TF) pig is a locally developed new breed resulting from 15 years of joint breeding efforts by Sichuan Techlex Co., Ltd., Sichuan Agricultural University, and Sichuan Provincial Animal Husbandry Station (11). As a new variety bred in 2011 in China, TF pigs were obtained by crossing traditional Chinese indigenous pig breeds with modern breeds {Duroc × [Landrace × (York × Meishan)]} (12), which has the characteristics of high meat production performance (lean meat percentage > 63.0 %), good reproductive performance (average litter size > 13.0), and excellent meat quality (intramuscular fat > 2.2 %).

In recent years, despite significant advancements in genetic breeding, nutritional levels, and feeding environments enhancing the growth performance of pigs, meat quality has deteriorated (13–19). Genetic selection focuses more on improving the reproductive capacity of sows while neglecting meat quality (20). Faster-growing pigs exhibit poorer stress resistance and are more prone to developing pale soft exudative (PSE) meat, particularly in the summer (21). Moreover, the accelerated growth rates of pigs lead to insufficient deposition of intramuscular fat (IMF) and nutrients that affect flavor, resulting in decreased tenderness and juiciness of pork (22). Amino acids are not only key nutrients in meat but also an important class of flavor compounds (23, 24). However, as water-soluble compounds, the content and proportion of amino acids are easily influenced by various factors, such as animal species, sex, SW, postmortem treatment, and storage conditions (23, 25–27). Previous studies have also found that an increase in SW is closely related to enhanced flavor, juiciness, and overall acceptability of pork (28, 29). For example, pork from pigs weighing 120 kg exhibited higher levels of most unsaturated fatty acids and total polyunsaturated fatty acid esters in the *longissimus dorsi* (*LD)* muscle compared to those in low-weight groups (110 kg and 100 kg). The SW also significantly affected the content of various flavor compounds. However, the amount of flavor compounds (aldehydes) derived from fatty acid oxidation was also significantly higher compared to other groups (28). Another study showed that an increase in SW up to 130 kg in pigs improved some aspects of carcass quality essential for the Teruel ham industry (30), especially with a significant improvement in meat color (higher a^*^ value) (31) or meat flavor (32).

Omics technologies are widely used to evaluate meat quality (33). Metabolomics can be used to analyze the changes in the overall metabolite content and levels in biological samples (34). This approach allows a comprehensive qualitative and quantitative analysis of metabolites to clarify how animals deal with the effects of environmental factors at the molecular level (34). Metabolomics is widely used in meat quality research to directly evaluate the effects of meat quality on human health (34), including the mechanisms of meat amino acid composition (34), meat color, and meat flavor (23, 24).

However, to date, it is unclear how different SW would affect the quality of TF pork, and there has been no research reported on the effects of SW on amino acids in the *LD* muscle of TF pigs. It is hypothesized that increasing SW might increase the IMF content and improve meat quality. Accordingly, in this study, metabolomics [high-performance liquid chromatography-mass spectrometry (HPLC-MS/MS) analysis] was employed to investigate the effects of different SW on the *LD* muscles and the regulatory mechanism of SW on the pork quality of TF pigs, providing theoretical support for enhancing the meat quality of TF pigs through genetic breeding.

## 2 Materials and methods

### 2.1 Experimental design and animal management

A total of 300 healthy male TF pigs weighing 70.16 ± 1.08 kg and aged 130 days, purchased from Techlex Food Co. Ltd. (Mianyang, China), were raised in a pig farm with cement flooring under captive feeding (15 pigs per pen and 1.3 cm^2^ per pig), and they had access to water *ad libitum*. For the feeding regime, we adhered to the pig farm's recommended feed formula and feeding mode (35). Based on SW quantity, 13 pigs were divided into three groups (100-kg group, 125-kg group, and 150-kg group with an average weight of 101.35 ± 1.26 kg, 123.46 ± 1.17 kg, and 151.54 ± 0.68 kg, respectively) with 3, 5, and 5 pigs in each group, respectively.

### 2.2 Sample preparation

Approximately 50 g of *LD* muscles were collected from the 10th rib of the left side carcass, frozen in liquid nitrogen, and sent to Metware Biotechnology Co. Ltd. (Wuhan, China) for LC-MS/MS metabolomics analysis. The *LD* muscles from the 10th ribs were used to detect the pH value, meat color, drip loss, cooking loss, and shear force.

### 2.3 Carcass traits of TF pigs

Carcass weight was recorded to calculate the dressing percentage. The values of backfat thickness at the first rib, last rib, and last lumbar were recorded to calculate the average backfat depth. The loin eye area (LEA) was measured at the last lumbar.

### 2.4 Meat quality of TF pigs

The muscle pH value and meat color (lightness L^*^, redness a^*^, and yellowness b^*^) were measured at 45 min and 24 h postmortem using a pH meter (pH-STAR, SFK-Technology, Denmark) and a portable chroma meter (CR-300, Minolta, Japan), respectively. The drip loss percentage was detected as previously described (36). Cooking loss was expressed as the weight change percentage (37). Shear force was detected using a texture analyzer (TA.XT Plus, Stable Micro Systems, Godalming, UK). The IMF was measured according to the national standard method (GB 5009.6-2016, China) (38).

### 2.5 LC-MS/MS metabolomics analysis of the *LD* muscles

#### 2.5.1 *LD* muscle sample preparation for metabolomics

The *LD* muscle samples of 13 TF pigs from three treatments were collected for LC-MS metabolomics analysis. Approximately 50 mg of frozen muscles were homogenized in 500 μL of methanol aqueous solution (70%, precooled at −20°C), whirled for 3 min, and then centrifuged at 12,000 × g for 10 min at 4°C. The supernatant (300 μL) was transferred to Eppendorf (EP) tubes, kept in a refrigerator at −20°C for 30 min, and then centrifuged at 12,000 × g for 10 min at 4°C. Then, the remaining supernatant (200 μL) was transferred to an LC-MS sampling vial with an inner liner for LC-MS analysis.

The stability of the LC-MS/MS system was monitored using five quality control samples within the analysis sequence, which were prepared from the pooled *LD* muscle samples.

#### 2.5.2 Metabolomics data capture

An ultraperformance liquid chromatography system was used to carry out the chromatographic separation of the *LD* muscles (QTRAP 6500+, SCIEX, USA). The *LD* muscle samples were injected into the ACQUITY BEH Amide column (100 × 2.1 mm, 1.7 μm) at 40°C (0.40 mL/min flow rate). The optimal linear gradient program was based on the description provided by Li and Shen (39), and the optimal mobile phase included ultra-pure water (containing 2 mM ammonium acetate and 0.04 % formic acid) in water (A) and acetonitrile (containing 2 mM ammonium acetate and 0.04 % formic acid) in water (B).

HPLC-MS/MS (QTRAP 6500+, SCIEX, USA), fitted with a dual electrospray ionization source (ESI) operating in positive and negative ion modes, was used to acquire mass data. The scan time was set at 5 spectra/s, and the centroid mode was from 50 to 1,000 m/z. The optimal conditions of analysis were based on the description provided by Li and Shen (39).

#### 2.5.3 Multivariate statistical analysis

Compound Discoverer 3.0 (Thermo Fisher, USA) was used to convert the raw MS spectra to a common data format (.mzML). Candidate metabolites [Variable importance for the projection (VIP) > 1 and an adjusted *P*-value of <0.05] were regarded as potential biomarkers. Further details of the metabolomics analysis are provided in Supplementary material 1.

#### 2.5.4 Identification of amino acid metabolism profiles and pathway analysis

The metabolite structure was confirmed through the LC-MS/MS analysis. METLIN was used to search for an accurate mass value of the amino acid metabolites and MS/MS fragment ions. The KEGG and HMDB databases were used to search for metabolic pathways and biochemical reactions. Pathway analysis and visualization were conducted using Compound Discoverer 3.0 (Thermo Fisher Scientific) software.

### 2.6 Statistical analysis

All data were analyzed using a one-way analysis of variance (ANOVA) (Statistical Package for the Social Sciences, SPSS, version 23.0, Inc., Chicago, IL, USA). The results are presented as the standard error of the means (SEM) for biological replicates. Significance was determined at a *p*-value of <0.05, and extreme significance was set at a *p*-value of <0.01.

## 3 Results

### 3.1 Meat quality of TF pigs

The carcass weight (*P* < 0.01), dressing percentage (*P* < 0.01), and loin eye area (LEA) (*P* < 0.01) were all higher in pigs in the 150-kg group compared to those in the 125-kg and 100-kg groups (Table 1). Additionally, the carcass length (*P* < 0.01) and backfat thickness (*P* < 0.01) were higher in 150-kg and 125-kg pigs than in 100-kg pigs.

**Table 1:** Carcass characteristics of TF pigs in the three SW groups.

**Items**	**100-kg group**	**125-kg group**	**150-kg group**	**SEM**	***P*-value**
*n*	3	5	5	-	-
SW (kg)	101.35^c^	123.46^b^	151.54^a^	5.68	<0.01
Hot carcass weight (kg)	74.80^c^	91.44^b^	112.96^a^	4.33	<0.01
Dressing percentage (%)	73.80^b^	74.06^b^	74.54^a^	0.10	<0.01
Carcass length (cm)	92.33 ^b^	100.36^a^	107.33^a^	2.06	<0.01
LEA (cm^2^)	38.05^b^	39.97^b^	42.63^a^	0.66	<0.01
Average backfat thickness (mm)	25.23^b^	36.54^a^	45.96^a^	2.86	<0.01

Different letters in the same row represent significant differences according to the ANOVA test. SW, slaughter weight; LEA, loin eye area.

The meat quality traits of the *LD* muscles in TF pigs are shown in Table 2. Compared with the 100-kg group, the 125-kg and 150-kg groups exhibited a decrease (*P* < 0.01) in shear force and an increase (*P* < 0.01) in the IMF content.

**Table 2:** Meat quality attributes of TF pigs in the three SW groups.

**Items**	**100-kg group**	**125-kg group**	**150-kg group**	**SEM**	***P-*value**
*n*	3	5	5	-	*-*
pH_45min_	6.73	6.57	6.57	0.05	0.369
pH_24h_	5.73	5.82	5.76	0.04	0.669
**Meat color**					
${\text{L}}_{45\text{min}}^{*}$	37.09^a^	33.31^b^	34.00^ab^	0.69	0.088
${\text{a}}_{45\text{min}}^{*}$	2.58	2.92	3.08	0.11	0.248
${\text{b}}_{45\text{min}}^{*}$	2.36^a^	1.99^ab^	1.67^b^	0.11	0.058
${\text{L}}_{24\text{h}}^{*}$	36.16	35.12	35.45	1.08	0.946
${\text{a}}_{24\text{h}}^{*}$	3.31	4.06	3.80	0.16	0.185
${\text{b}}_{24\text{h}}^{*}$	3.27	3.85	3.98	0.14	0.137
Drip loss (%)	2.24	2.03	2.31	0.10	0.538
Cooking loss (%)	33.49	33.28	32.20	0.60	0.682
Shear force (N)	50.43^a^	39.71^b^	39.17^b^	1.70	<0.01
IMF content (%)	1.50^c^	2.52^b^	3.74^a^	0.28	<0.01

Different letters in the same row represent significant differences according to the ANOVA test. IMF, intramuscular fat.

### 3.2 *LD* metabolic responses in the TF pigs with different SW

**Figure 1:**
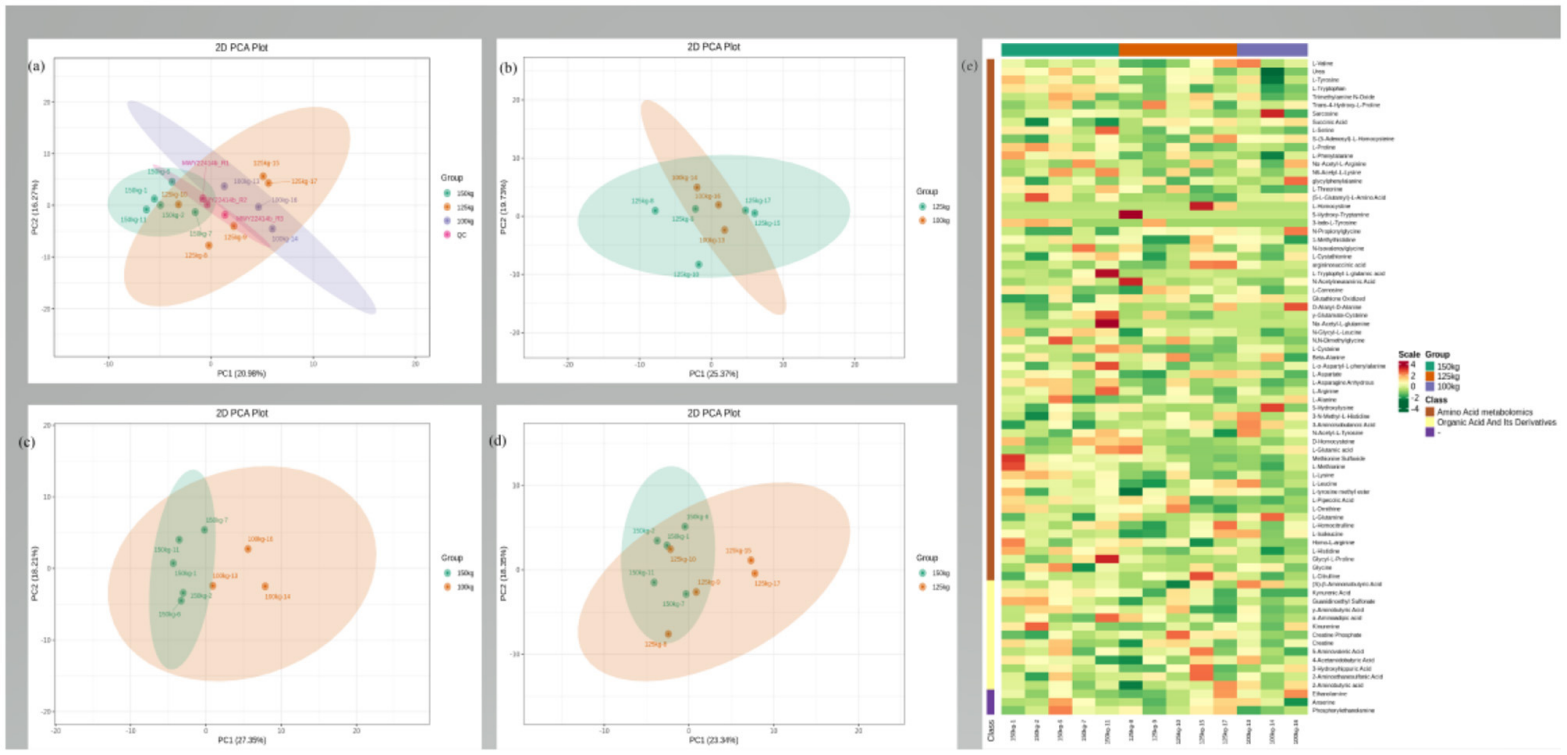
Muscle metabolome analysis. **(a–d)** Principal component analysis (PCA) of the *LD* muscle metabolites from 100-kg (*n* = 3), 125-kg (*n* = 5), and 150-kg (*n* = 5) TF pigs. **(a)** All groups; **(b)** 125-kg group vs. 100-kg group; **(c)** 150-kg group vs. 100-kg group; **(d)** 150-kg group vs. 125-kg group. **(e)** Hierarchical cluster analysis of the metabolome from the *LD* muscle of TF pigs. Heat map representation of metabolites that differed significantly between *LD* muscle samples of three SW of TF pigs. Each block represents the abundance of one metabolite in one sample.

**Figure 2:**
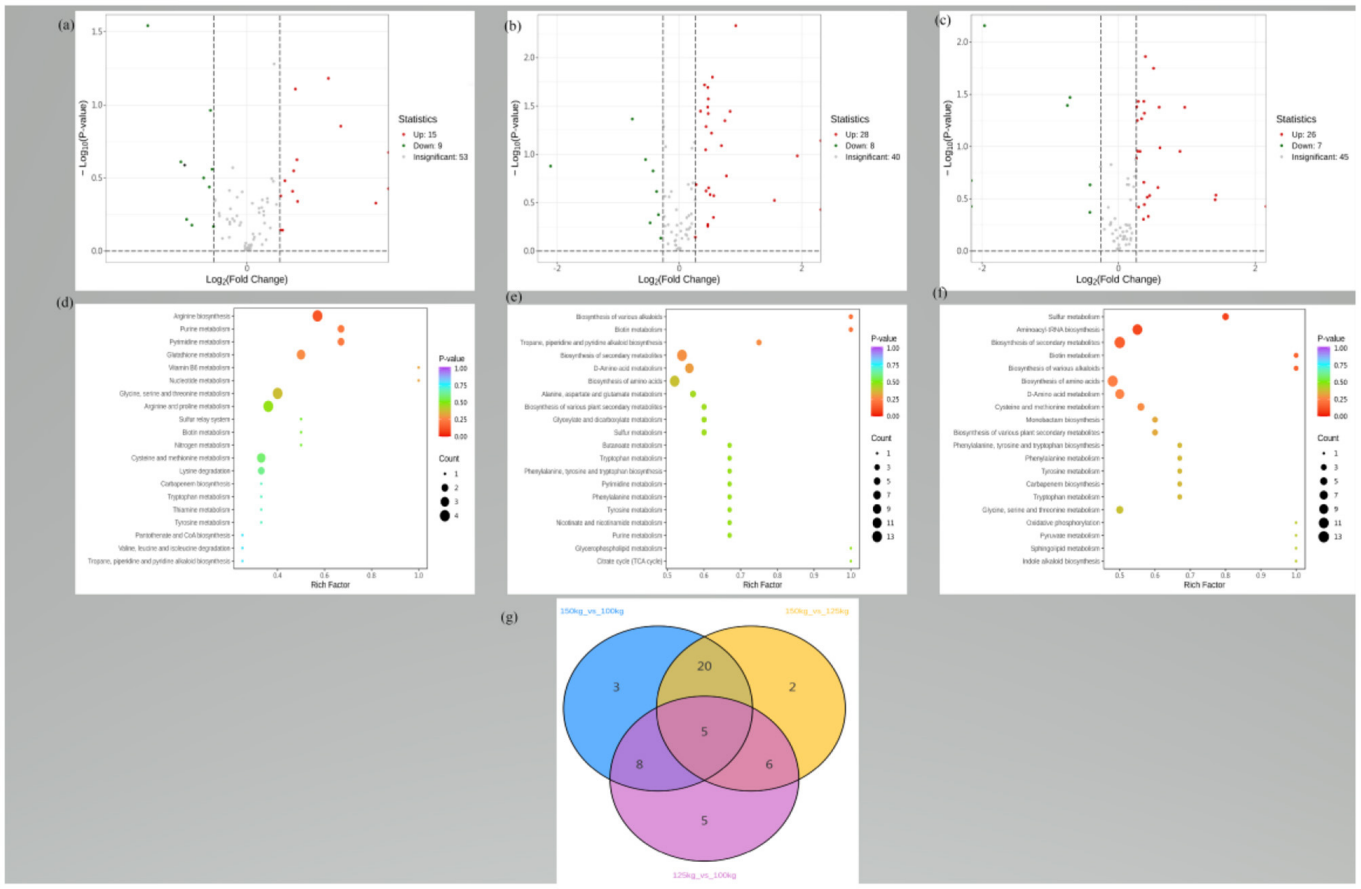
**(a–c)** Volcano plot representing the significant variables in the discrimination of *LD* muscle metabolites from three body-weight TF pigs. **(a)** Number of DEMs in the 125-kg group vs. 100-kg group comparison. **(b)** Number of DEMs in the 150-kg group vs. 100-kg group comparison. **(c)** Number of DEMs in the 150-kg group vs. 125-kg group comparison. Gray, metabolites have an unchanged abundance. Red, upregulated metabolites in the 125-kg group **(a)**, the 150-kg group **(b)**, and the 150-kg group **(c)**. Blue, downregulated metabolites in the 125-kg group **(a)**, the 150-kg group **(b)**, and the 150-kg group **(c)**. Red and blue squares in volcano plots are model-separated metabolites following the conditions of (VIP) >1 and a *p*-value of *t*-test <0.05 and fold change (FC) ≥1.2, or FC ≤ 0.833. **(d–f)** The DEMs were clarified by the KEGG pathways, and the pathways of the 125-kg group vs. 100-kg group, 150-kg group vs. 100-kg group, and 150-kg group vs. 125-kg group comparisons were displayed. **(d)** Topology analysis of the metabolic pathways identified in the *LD* muscle (*n* = 20) metabolites of TF pigs from the 125-kg and 100-kg groups; **(e)** Topology analysis of the metabolic pathways identified in the *LD* muscle metabolites of TF pigs from the 150-kg and 100-kg groups; **(f)** Topology analysis of the metabolic pathways identified in the *LD* muscle metabolites of TF pigs from the 150-kg and 125-kg groups. The advanced bubble chart shows the enrichment of DEMs in signaling pathways. The x-axis represents the rich factor (rich factor = number of DEMs enriched in the pathway/number of all metabolites in the background metabolites set). The y-axis represents the enriched pathway. The size of the bubble represents the number of DEMs enriched in the pathway, and the color represents enrichment significance. **(g)** Venn diagrams of DEMs from the 125-kg, 100-kg, and 150-kg group comparisons were displayed. Each circle in the figure represented a comparison group, and the number shared between circles represented the number of DEMs shared between the comparison groups. The number without overlap represented the number of DEMs unique to the comparison group.

The typical total ion chromatograms of *LD* samples through the HPLC-MS/MS analysis displayed good separation, peak shape, and strong intensity. The well-fitting principal component analysis (PCA) models displayed clear separations between the three groups (Figures 1a–d). According to the VIP value (>1) and *P*-value (<0.05), 93 DEMs were identified, including 69 upregulated DEMs and 24 downregulated DEMs (Figure 2). Hierarchical clustering analysis further distinguished the *LD* samples of the 125-kg and 150-kg groups from those of the 100-kg group (Figure 1e).

The DEMs of *LD* muscles obtained in the treatment groups were submitted to the KEGG website for relevant pathway analysis. The most important KEGG metabolic pathways were identified when comparing the 125-kg vs. 100-kg groups, 150-kg vs. 100-kg groups, and 150-kg vs. 125-kg groups (Figures 2d–f). SW significantly altered the metabolic pathways. The predominant metabolites were arginine and proline metabolism, glycine, serine and threonine metabolism, alanine, aspartate and glutamate metabolism, tryptophan metabolism, and beta-alanine metabolism. The details of the top 12 *LD* muscle metabolites between different groups are shown in Table 3. A Venn diagram comparing DEMs for the three groups revealed that there were 24, 36, and 33 DEMs between 125-kg vs. 100-kg groups, 150-kg vs. 100-kg groups, and 150-kg vs. 125-kg groups, respectively. Furthermore, five DEMs were the most common among all three comparisons (Figure 2g). The detailed information on 93 DEMs identified across three comparisons is shown in Table 4. Urea, 3-iodo-L-tyrosine, N-glycyl-L-leucine, N,N-dimethylglycine, and kynurenic acid might be potential biomarkers for the three SW comparisons.

**Table 3:** Top 12 amino acid metabolites of *LD* muscles in the TF pigs.

**No**.	**Ionization model**	***m/z***	**Formula**	**Metabolites**	**Ion mode**	**Fold change**	***P*-value**
1	[M-H]^−^	118.03	C_4_H_6_O_4_	Succinic-acid	Negative	0.98847	0.92353
2	[M-H]^−^	131.06	C_5_H_9_NO_3_	N-propionylglycine	Negative	0.90677	0.80671
3	[M-H]^−^	159.09	C_7_H_13_NO_3_	N-isovaleroylglycine	Negative	1.11198	0.83821
4	[M-H]^−^	125.15	C_2_H_7_NO_3_S	2-Aminoethanesulfonic-acid	Negative	1.07024	0.54600
5	[M+H]^+^	612.15	C_20_H_32_N_6_O_12_S_2_	Glutathione-oxidized	Positive	0.91251	0.51476
6	[M+H]^+^	384.12	C_14_H_20_N_6_O_5_S	S-(5-Adenosyl)-L-homocysteine	Positive	0.90740	0.38512
7	[M+H]^+^	333.13	C_16_H_19_N_3_O_5_	TRP-GLU	Positive	1.10202	0.64443
8	[M+H]^+^	309.11	C_11_H_19_NO_9_	N-acetylneuraminic-acid	Positive	1.28692	0.38958
9	[M+H]^+^	306.97	C_9_H_10_INO_3_	3-Iodo-L-tyrosine	Positive	Inf	0.37390
10	[M+H]^+^	290.27	C_10_H_18_N_4_O_6_	Argininosuccinic-acid	Positive	1.13755	0.69561
11	[M+H]^+^	280.11	C_13_H_16_N_2_O_5_	Asp-Phe	Positive	0.99002	0.96465
12	[M+H]^+^	268.06	C_8_H_16_N_2_O_4_S_2_	L-homocystine	Positive	Inf	0.21155

RT, retention time.

**Table 4:** Significantly altered abundance of *LD* muscle metabolites in the TF pigs of different SW groups.

**Items**	**Metabolites**	**Class**	**Type**	**FDR**	***P*-value**
**125 kg vs. 100 kg (up 15, down 9)**					
	Urea	Amino acid metabolomics	Up	0.97984	0.23734
	Trimethylamine-N-Oxide	Amino acid metabolomics	Up	0.97984	0.71889
	L-Homocystine	Amino acid metabolomics	Up	0.97984	0.21155
	5-Hydroxy-Tryptamine	Amino acid metabolomics	Up	0.97984	0.37390
	3-Iodo-L-Tyrosine	Amino acid metabolomics	Up	0.97984	0.37390
	L-Cystathionine	Amino acid metabolomics	Up	0.97984	0.72041
	N-Acetylneuraminic-Acid	Amino acid metabolomics	Up	0.97984	0.38958
	N-Glycyl-L-Leucine	Amino acid metabolomics	Up	0.97984	0.28271
	N, N-Dimethylglycine	Amino acid metabolomics	Up	0.97984	0.45714
	L-Pipecolic-Acid	Amino acid metabolomics	Up	0.97984	0.13995
	L-Ornithine	Amino acid metabolomics	Up	0.97984	0.33016
	L-Citrulline	Amino acid metabolomics	Up	0.97984	0.07777
	Kynurenic-Acid	Organic acid and its derivatives	Up	0.97984	0.46930
	Creatine-Phosphate	Organic acid and its derivatives	Up	0.97984	0.06576
	5-Aminovaleric-Acid	Organic acid and its derivatives	Up	0.97984	0.41903
	Ethanolamine	N/A	Down	0.97984	0.36436
	Sarcosine	Amino acid metabolomics	Down	0.97984	0.66537
	Nα-Acetyl-L-Arginine	Amino acid metabolomics	Down	0.97984	0.60772
	Glycylphenylalanine	Amino acid metabolomics	Down	0.97984	0.68025
	(5-L-Glutamyl)-L-Amino-Acid	Amino acid metabolomics	Down	0.97984	0.02872
	Cys	Amino acid metabolomics	Down	0.97984	0.31605
	N-Acetyl-L-Tyrosine	Amino acid metabolomics	Down	0.97984	0.24547
	Gln	Amino acid metabolomics	Down	0.97984	0.27582
	(S)-β-Aminoisobutyric-Acid	Organic acid and its derivatives	Down	0.97984	0.10905
**150 kg vs. 100 kg (up 28, down 8)**					
	Urea	Amino acid metabolomics	Up	0.34102	0.08133
	Trp	Amino acid metabolomics	Up	0.32806	0.06043
	Trimethylamine-N-Oxide	Amino acid metabolomics	Up	0.76478	0.53255
	Ser	Amino acid metabolomics	Up	0.30224	0.02031
	Pro	Amino acid metabolomics	Up	0.30224	0.03585
	Phe	Amino acid metabolomics	Up	0.34102	0.08974
	Thr	Amino acid metabolomics	Up	0.30224	0.02664
	3-Iodo-L-Tyrosine	Amino acid metabolomics	Up	0.34102	0.07223
	1-Methylhistidine	Amino acid metabolomics	Up	0.73197	0.45015
	N-Isovaleroylglycine	Amino acid metabolomics	Up	0.85243	0.71905
	L-Cystathionine	Amino acid metabolomics	Up	0.76478	0.55346
	Trp-Glu	Amino acid metabolomics	Up	0.58159	0.29845
	γ-Glutamate-Cysteine	Amino acid metabolomics	Up	0.55709	0.23868
	Nα-Acetyl-L-glutamine	Amino acid metabolomics	Up	0.69490	0.37390
	N-Glycyl-L-Leucine	Amino acid metabolomics	Up	0.30224	0.04495
	N, N-Dimethylglycine	Amino acid metabolomics	Up	0.50791	0.16708
	Asp-Phe	Amino acid metabolomics	Up	0.56364	0.26730
	Asn	Amino acid metabolomics	Up	0.30224	0.01589
	D-Homocysteine	Amino acid metabolomics	Up	0.55709	0.22179
	Methionine-Sulfoxide	Amino acid metabolomics	Up	0.30224	0.05170
	Met	Amino acid metabolomics	Up	0.30224	0.03601
	Lys	Amino acid metabolomics	Up	0.30224	0.01913
	L-Pipecolic-Acid	Amino acid metabolomics	Up	0.30224	0.00467
	L-Ornithine	Amino acid metabolomics	Up	0.30224	0.03238
	His	Amino acid metabolomics	Up	0.30224	0.03794
	Glycyl-L-Proline	Amino acid metabolomics	Up	0.56364	0.26071
	Kynurenic-Acid	Organic acid and its derivatives	Up	0.37565	0.10380
	γ-Aminobutyric-Acid	Organic acid and its derivatives	Up	0.55709	0.20536
	Ethanolamine	N/A	Down	0.55709	0.24190
	Succinic-Acid	Amino acid metabolomics	Down	0.30224	0.04319
	Nα-Acetyl-L-Arginine	Amino acid metabolomics	Down	0.85243	0.73572
	Glycylphenylalanine	Amino acid metabolomics	Down	0.76478	0.51163
	N-Propionylglycine	Amino acid metabolomics	Down	0.43750	0.13240
	Glutathione-Oxidized	Amino acid metabolomics	Down	0.39124	0.11325
	D-Alanyl-D-Alanine	Amino acid metabolomics	Down	0.73197	0.42181
	Gln	Amino acid metabolomics	Down	0.47010	0.14845
**150 kg vs. 125 kg (up 26, down 7)**					
	Urea	Amino acid metabolomics	Up	0.32770	0.03694
	Trp	Amino acid metabolomics	Up	0.32770	0.01376
	Ser	Amino acid metabolomics	Up	0.32770	0.03690
	Pro	Amino acid metabolomics	Up	0.33843	0.05420
	Phe	Amino acid metabolomics	Up	0.32770	0.04157
	Thr	Amino acid metabolomics	Up	0.54810	0.12971
	(5-L-Glutamyl)-L-Amino-Acid	Amino Acid metabolomics	Up	0.32770	0.04201
	3-Iodo-L-Tyrosine	Amino acid metabolomics	Up	0.69529	0.29158
	1-Methylhistidine	Amino acid metabolomics	Up	0.84255	0.46735
	TRP-GLU	Amino acid metabolomics	Up	0.69854	0.32225
	γ-Glutamate-Cysteine	Amino acid metabolomics	Up	0.69854	0.30526
	Nα-Acetyl-L-glutamine	Amino acid metabolomics	Up	0.72656	0.37390
	N-Glycyl-L-Leucine	Amino acid metabolomics	Up	0.62007	0.21993
	N, N-Dimethylglycine	Amino acid metabolomics	Up	0.84255	0.49689
	Cys	Amino acid metabolomics	Up	0.51085	0.10288
	Asp-Phe	Amino acid metabolomics	Up	0.62007	0.24644
	Asn	Amino acid metabolomics	Up	0.33843	0.04812
	Arg	Amino acid metabolomics	Up	0.51085	0.110817
	N-Acetyl-L-Tyrosine	Amino acid metabolomics	Up	0.72656	0.37948
	D-Homocysteine	Amino acid metabolomics	Up	0.72658	0.35846
	Methionine-Sulfoxide	Amino acid metabolomics	Up	0.51085	0.11134
	Met	Amino acid metabolomics	Up	0.32770	0.04193
	Lys	Amino acid metabolomics	Up	0.32770	0.01783
	His	Amino acid metabolomics	Up	0.33843	0.05640
	Glycyl-L-Proline	Amino acid metabolomics	Up	0.69529	0.29416
	Kynurenic-Acid	Organic acid and its derivatives	Up	0.51085	0.11122
	Succinic-Acid	Amino acid metabolomics	Down	0.32770	0.04031
	L-Homocystine	Amino acid metabolomics	Down	0.62007	0.21155
	5-Hydroxy-Tryptamine	Amino acid metabolomics	Down	0.72658	0.37390
	N-Propionylglycine	Amino acid metabolomics	Down	0.32770	0.00698
	Argininosuccinic-acid	Amino acid metabolomics	Down	0.79207	0.42650
	Glutathione-Oxidized	Amino acid metabolomics	Down	0.62007	0.23257
	Creatine-Phosphate	Organic acid and its derivatives	Down	0.32770	0.03382

## 4 Discussion

Target SW, age at slaughter, and fat deposition rate might lead to differences in carcass characteristics. In this study, carcass characteristics (dressing percentage, backfat thickness, carcass length, and LEA) of TF pigs differed significantly with increasing SW, especially in the 150-kg group, which was consistent with previous findings from studies conducted between 100 to 160 kg (40) or from 110 to 150 kg (41). However, another study found that backfat thickness was not affected by SW (from 145 to 156 kg) (42), which might be because the size of adipocytes increased with age, and as animals grew, the lipid content increased (42). Once the cells were filled with fat, any increase in thickness was not as significant (42). These results confirmed that the levels of most carcass characteristics were affected by SW. However, the values of pH_45min_, pH_24h_, ${\text{a}}_{45\text{min}}^{*}$, ${\text{L}}_{24\text{h}}^{*}$, ${\text{a}}_{24\text{h}}^{*}$, ${\text{b}}_{24\text{h}}^{*}$, drip loss, or cooking loss of *LD* muscles did not differ between the treatments.

IMF is an important flavor precursor that significantly affects the juiciness, tenderness, and overall flavor of meat (43). It plays a vital role in improving meat tenderness, as the texture of meat is mainly determined by the myofibrils and connective tissues. IMF is generally found on the outer membrane, within the fasciculus, and in the endometrium of intramuscular fibers. Consequently, a higher density of muscle fibers can lead to greater IMF deposition. The presence of IMF causes the connective tissues to become less dense, reducing their interaction with muscle fibers. This makes the tissue easier to separate, thus improving meat tenderness. In the present study, IMF content increased significantly with increasing SW, which was similar to the previous studies (25). Generally, an IMF content of 2–3% is considered ideal for fresh meat quality. In this study, the IMF content of the *LD* muscles from the 125-kg and 150-kg groups was recorded at 2.5% and 3.7%, respectively, which indicated that increasing SW might improve meat flavor. Lipid variations (five DEMs including decanoic acid, hexanoic acid, octanoic acid, nervonic acid, and erucic acid) (unpublished data) in the muscle tissues between the three groups could be closely associated with fat deposition rates during the animals' growth and development stages. The fatty acid degradation pathway was downregulated, and IMF deposition was increased in the later stages (98–140 days) (44).

Shear force is a key determinant of meat tenderness; lower shear force correlates with greater tenderness. IMF significantly influences shear force; it can decrease the density and mechanical structure of connective tissues, facilitating the separation of muscle tissues and reducing muscle shear force, thereby improving muscle tenderness. This study found that increasing SW could significantly reduce shear force, thereby improving tenderness.

In the present study, metabolomics was used to explore how SW affects the meat quality of TF pigs. We identified 93 DEMs across three SW groups. The separation of *LD* muscle samples from the three groups observed in the PCA indicated that SW affected the amino acid metabolomic profiles of *LD* muscles. Our results confirmed that the levels of most metabolites were affected by SW. The results of the metabolic pathway analysis indicated that the mechanism of SW might be chiefly related to metabolic pathway metabolism. Furthermore, five DEMs have been identified as potential biomarkers for distinguishing among the three SW groups.

Amino acids are a key indicator of protein nutrition and one of the main factors affecting pork freshness. During pork flavor formation, fresh amino acids, such as glycine and glutamate, play an important role. Glycine and glutamate are typical representatives of antioxidant-related amino acids. When the essential amino acid content of proteins in meat is high, it is beneficial for enhancing the human immune system. Aromatic amino acids, such as tyrosine and tryptophan, play a crucial role in the metabolic pathways of animal bodies. Glycine is a non-essential amino acid component of reduced glutathione, an endogenous antioxidant. It is often externally supplemented when the body experiences severe stress. A previous study showed that dietary supplementation of glycine in a low-protein diet could be used to improve meat quality (45). Pigs with intrauterine growth restriction (IUGR) have suboptimal growth performance and impaired glycine synthesis. Dietary glycine supplementation greatly increased the meat a^*^ value of IUGR pigs by 10% (46). Glutamine (Gln), a precursor for glutamic acid, is a non-essential amino acid. L-glutamine is a coding amino acid in protein synthesis. A previous study indicated that Gln supplementation in broiler diets might alleviate heat stress-caused deterioration in meat quality and meat color stability (47). Tyrosine (Tyr), an aromatic polar α-amino acid containing phenolic and hydroxyl groups, is one of the conditionally essential amino acids for the human body. It was found that the intake of amino acids might be related to stress susceptibility induced by hormones and neurotransmitters. Dietary regulation of neurotransmitter amino acid precursors (Tyr) might reduce stress responses in pigs and decrease the incidence of pale, soft, and exudative pork (48). In the current study, the abundance of Tyr in the three TF pig comparisons was upregulated. Tryptophan (Trp) is one of the essential amino acids in the human body and a precursor to the important neurotransmitter serotonin (48). Dietary manipulation of Trp (amino acid precursors of neurotransmitters) may reduce stress responses in pigs and reduce the occurrence of PSE meat (48). As a sedative, it can regulate mental rhythms and improve sleep. Adding Trp to sheep diets reduced stress responses by enhancing the production of 5-HT from the nervous system, which improved meat quality (26). In this study, the abundance of Trp in three TF pig comparisons was upregulated. Upregulated Trp in the three comparisons of TF pigs indicated that increasing SW might enhance the stress resistance of TF pigs and improve meat quality.

Chemical and metabolomic analyses showed that, with the increase in SW, IMF deposition improved, and the abundance of tryptamine, Tyr, Gln, propionylglycine, and the metabolites in the *LD* muscles also increased. With increasing SW, the synthesis of multiple non-essential and essential amino acids in the *LD* muscle was also enhanced, which might contribute to the improvement in meat quality.

## 5 Conclusion

This study focused on the mechanism by which SW affected the meat quality of TF pigs. The metabolomics analysis showed that 93 DEMs were significantly enriched in pathways related to amino acid metabolism, such as arginine and proline metabolism, alanine, aspartate and glutamate metabolism, tryptophan metabolism, and beta-alanine metabolism. In general, increasing SW was found to improve the pork quality of TF pigs (reduced shear force of *LD* muscles and increased IMF content of *LD* muscles). However, the negative impact of increased SW on backfat thickness warrants further consideration. It can be concluded that a SW of 125 kg is more economical.

## Data availability statement

The original contributions presented in the study are included in the article/Supplementary material, further inquiries can be directed to the corresponding author.

## Ethics statement

The animal study was approved by the Institutional Animal Care and Use Committee of the Yibin University. The study was conducted in accordance with the local legislation and institutional requirements.

## Author contributions

YL: Conceptualization, Data curation, Formal analysis, Investigation, Methodology, Resources, Software, Writing – original draft. XT: Formal analysis, Investigation, Resources, Writing – review & editing. PZ: Conceptualization, Supervision, Writing – review & editing. JZ: Conceptualization, Funding acquisition, Supervision, Writing – review & editing. XA: Conceptualization, Funding acquisition, Methodology, Project administration, Resources, Supervision, Writing – review & editing.
