# Supplementary material for: Effects of slaughter weight on carcass characteristics, meat quality, and metabolomics profiling in the longissimus dorsi muscle of Tianfu finishing pigs
Source: Front Vet Sci. 2024 Jun 28;11:1420634. doi: 10.3389/fvets.2024.1420634 (PMC11239573; doi:10.3389/fvets.2024.1420634)
Supplement: Supplementary file 1 [file Table_1.DOCX]

**Metabolomics data acquisition**

The optimal linear gradient program was shown as followed: 0–1.2min, A:B= 10:90 (v/v); 1.3–9.0 min, A:B= 40:60 (v/v); 10.0–11.0 min, A:B= 60:40 (v/v); 11.01–15.0 min, A:B= 10:90 (v/v). Post time was set to 3 min for equilibration.

The scan time was set at 5 spectra/s and the data was collected in centroid mode from 50 to 1000 m/z. The optimal conditions of analysis were as followed: desolvation gas rate was set to 0.4 L/min at 550 °C and the nebulizer pressure was set at 35 psi; the fragment voltage was 60 V in both modes, capillary voltages were 3.5 KV in positive and negative modes.

**Multivariate statistical analysis**

The obtained data sets were introduced into SIMCA-P 14.1 (Umetrics，Umea，Sweden) to perform unsupervised principal component analysis (PCA), supervised partial least squares discriminant analysis (PLS-DA), and supervised orthogonal partial least squares discriminant analysis (OPLS-DA). The fitness and predictive capability of the PCA, PLS-DA, and OPLS-DA models were evaluated by the cumulative R^2^ and Q_2_. Variable importance for projection (VIP) value and loading-plots were constructed from the OPLS. Student's t-test with false discovery rate (FDR) was employed for univariate analysis across groups. The R language software was used for one-dimensional statistical analysis such as volcano map analysis, Student's *t*-test, and multiple of variation analysis to find the differential metabolites related to SW.
